# Effects of Very Low Dose Fast Neutrons on Cell Membrane And Secondary Protein Structure in Rat Erythrocytes

**DOI:** 10.1371/journal.pone.0139854

**Published:** 2015-10-05

**Authors:** A. Saeed, Gehan A. Raouf, Sherif S. Nafee, Salem A. Shaheen, Y. Al-Hadeethi

**Affiliations:** 1 Physics Department, Faculty of Science, King Abdulaziz University, Jeddah, KSA; 2 Biochemistry Department, Faculty of Science, King Abdulaziz University, Jeddah, KSA; University of Maribor, SLOVENIA

## Abstract

The effects of ionizing radiation on biological cells have been reported in several literatures. Most of them were mainly concerned with doses greater than 0.01 Gy and were also concerned with gamma rays. On the other hand, the studies on very low dose fast neutrons (VLDFN) are rare. In this study, we have investigated the effects of VLDFN on cell membrane and protein secondary structure of rat erythrocytes. Twelve female *Wistar* rats were irradiated with neutrons of total dose 0.009 Gy (^241^Am-Be, 0.2 mGy/h) and twelve others were used as control. Blood samples were taken at the 0, 4th, 8th, and 12th days postirradiation. Fourier transform infrared (FTIR) spectra of rat erythrocytes were recorded. Second derivative and curve fitting were used to analysis FTIR spectra. Hierarchical cluster analysis (HCA) was used to classify group spectra. The second derivative and curve fitting of FTIR spectra revealed that the most significant alterations in the cell membrane and protein secondary structure upon neutron irradiation were detected after 4 days postirradiation. The increase in membrane polarity, phospholipids chain length, packing, and unsaturation were noticed from the corresponding measured FTIR area ratios. This may be due to the membrane lipid peroxidation. The observed band shift in the CH_2_ stretching bands toward the lower frequencies may be associated with the decrease in membrane fluidity. The curve fitting of the amide I revealed an increase in the percentage area of α-helix opposing a decrease in the β-structure protein secondary structure, which may be attributed to protein denaturation. The results provide detailed insights into the VLDFN effects on erythrocytes. VLDFN can cause an oxidative stress to the irradiated erythrocytes, which appears clearly after 4 days postirradiation.

## Introduction

Living cells are exposed to ionizing radiation in many situations and accidents such as natural sources like cosmic rays, nuclear disasters in our world like Chernobyl or Fukushima Daiichi disaster, occupational works, and medical centers. Nowadays, most of the advanced medical centers give their patients ionizing radiation for both diagnoses and therapy. The effect of the ionizing radiation on living cell depends on the cell type, the energy and radiation type, the linear energy transferred (LET), the dose, the dose rate, and the time of testing post exposure [1, 2].

Ionizing radiation induces oxidative stress that is implicated in the pathogenesis of many diseases [3]. Erythrocyte is highly susceptible to oxidative stress, due to exposure to oxygen flux and their high concentration of polyunsaturated fatty acids [4]. Thus, any alteration in its structure is currently viewed as a promising indicator of disease or morbidity [4, 5].

The interaction of ionizing radiation affects mainly water molecules within living tissues. This interaction is called indirect action [6] that produces free radicals such as ${\text{H}}^{\dot{\;-}}$ and ${\text{OH}}^{\dot{-}}$ [7, 8]. These free radicals in turn can attack cellular membrane, DNA, and proteins [9]. Free radicals initiate peroxidative chain reactions in unsaturated lipid [10]. The degree of oxidative damage depends on the balance between the oxidative stress and the efficiency of the antioxidant mechanism [11].

The effects of ionizing radiation on living cells have been reported extensively in the literatures. Most of these reports were mainly concerned with high doses greater than 0.01 Gy and mainly using gamma rays. This may be due to use of gamma radiation in most equipment's of imaging and nuclear therapy. In contrast, studies on very low dose fast neutrons (VLDFN) are rare [12]. Nowadays, neutrons are used in advanced medical centers as external fast beam neutron therapy and boron neutron capture therapy (BNCT). BNCT is a noninvasive therapeutic for treating locally malignant tumors in brain and neck [13]. The deposited energy into tissue depends on the linear energy transfer (LET). X-rays and protons produce low LET radiation, whereas neutrons produce high LET radiation. Low LET radiation can damage cells by generating reactive oxygen species. The advantages of neutrons are uncharged and damage cells by nuclear interactions. Malignant tumors tend to have low oxygen levels and thus can be resistant to low LET radiation [14]. Currently, Neutron therapy is applied in tens of centers worldwide. The considerable number of patients exceeding 15,000 until 1997 and their follow-up [15] recently led to the use of fast neutron therapy.

The present study was carried out to lay the foundation to understand the influence of VLDFN (0.009 Gy) of average energy (4.5 MeV) on the erythrocytes lipid membrane and proteins in vivo for zero up to 12 days postexposure time intervals. The responses of erythrocytes to the ionizing radiation were studied by Fourier transform infrared (FTIR) spectroscopy, which is a very sensitive technique to any structural changes in biological tissues and hematology disorders at sub-molecular levels [16, 17].

## Materials and Methods

### Experimental animals

Twenty-four female *Wistar* rats (weighing 220–270 g) at three months of age were used in this study. They were purchased from the Breeding Laboratory at the King Fahd Medical Research Center (KFMRC), King Abdulaziz University, Jeddah, Saudi Arabia. The animals were allowed to acclimatize for 15 days and adapted at artificial day-night rhythm of 12 h under standard vivarium conditions with a relative humidity of 70%. Animals were maintained in box cages (3 rats/cage). They were given food and water *ad libitum*. This study was approved by Experimental Animal Research and Ethics Committee, KFMRC, King Abdulaziz University. Animals were randomly divided into two groups, twelve were irradiated; three rats were irradiated at each time. Twelve rats were sham-irradiated as control. Each test was analyzed separately using three irradiated rats and three control rats at time intervals postirradiation (0 day, 4, 8, and 12 days postirradiation). Rats were anaesthetized under ether during blood collection then were sacrificed by cervical dislocation and spleens were dissected that will be a subject to another study.

### Irradiation

Very low dose rate was applied to the posterior portions of the rats from 185 GBq (5 Ci) ^241^Am-Be neutron source capsule X.14 (code AMN.24) (Amersham International PLC, Buckinghamshire, England). ^241^Am-Be neutron source emits spectrum neutrons from 0 up to 11 MeV [18, 19] and an average energy around 4.5 MeV [8, 20, 21]. The neutron emission of the source is 1.1 × 10^7^ n s^−1^ with tolerance of around 10 %. Dosimetry measurements were performed using a neutron monitor NM2 (Nuclear Enterprise, Edinburgh, UK). Neutron monitor NM2 consists mainly of thermal neutron detector surrounded by a polyethylene moderator with a boron trifluoride (BF_3_) detector. The rats were irradiated at a dose rate of 0.2 mGy/h with neutrons total dose 9 mGy. Gamma rays and other electromagnetic radiations emitting from the neutron source were shielded using lead that is a good shielding material for gamma rays and other electromagnetic radiations, whereas lead is a poor shielding for fast neutrons. The thickness of used lead in the shielding was 9.6 cm. Eight percentage of neutron dose was unavoidable contamination from gamma rays but this contributed only negligibly to the biological effect because of the higher relative biological effectiveness (RBE) of the neutrons [22].

### Isolation of erythrocytes

At time intervals postirradiation (0 day, 4, 8, and 12 days postirradiation), each rat of the tested groups was anaesthetized under ether. Peripheral blood samples (3 ml) were collected through the retroorbital plexus using capillary tubes with a diameter of 1.5 mm into 4 ml anticoagulant dipotassium ethylenediaminetetraacetic acid (K_2_EDTA) tubes. Within 1 h of collection, the samples were processed. Blood samples were applied to Histopaque 1077 gradients (Sigma-Aldrich, Missouri, USA) following the manufacturer's protocol to separate plasma, mononuclear, and erythrocytes. Here, Histopaque 1077 gradient was used to separate peripheral blood mononuclear cells from the blood that will be a subject to another study. Blood samples with Histopaque 1077 gradients were centrifuged at 400 *x g* for 30 minutes at room temperature. After first centrifugation, the plasma was removed, and the mononuclear cells were separated; erythrocytes were washed three times with sodium phosphate buffered saline (SPB) (137 mM NaCl, 2.7 mM KCl, 10 mM Na_2_HPO_4_.2H_2_O, 2.0 KH_2_PO_4_, and pH 7.4) [23] and the buffy coat was discarded each time. The washing centrifugations were applied at 280 *x g* for 12 min (4°C) [24]. Then, all samples were stored at -80°C in a deep freeze. After that, the samples were lyophilized using ALPHA 1–2 LD_plus_ freeze dryer (Martin Christ Gefriertrocknungsanlagen GmbH, Osterode, Germany) at −60°C and under the vacuum 66 × 10^−3^ mbar [11, 25].

### FTIR spectra

Lyophilized erythrocytes with potassium bromide (KBr) (BDH Chemicals Ltd, Poole, England) were prepared as KBr discs. For each disc, 2 mg of lyophilized erythrocytes were ground to fine powder with mortar and pestle. 198 mg of KBr was then added with further grinding and mixing. The mixture was then pressed in a die at 6 metric tons force for 60 s [11, 26] to turn out to a clear transparent disc of diameter 13 mm and thickness 0.57 mm. At room temperature, FTIR spectra were recorded using a Shimadzu FTIR-8400S spectrophotometer (Shimadzu Corporation, Tokyo, Japan) with germanium-coated KBr plate beam splitter and temperature controlled high sensitivity deuterated, L-alanine doped triglycine sulfate (DLATGS) detector. The spectrometer was continuously purged with dry nitrogen to minimize atmospheric water vapor and carbon dioxide interference. Typically, 20 scans were signal-averaged for a single spectrum and at spectral resolution of 4 cm^−1^. The triplicate FTIR spectra were obtained from each rat. The spectra were collected with IRsolution software (Shimadzu Corporation, Tokyo, Japan). Then, they were saved as the joint committee on atomic and molecular physical data- data exchange (JCAMP-DX) format. After that, they were operated with OMNIC 8.3 software (Thermo Fisher Scientific Inc., Massachusetts, USA). To minimize the difficulties arising from unavoidable shifts, the entire spectrum has been baseline corrected in the 4000–400 cm^−1^ region. Every spectrum has been min–max normalized by scaling the entire spectrum to the absorbance of amide I (around 1654 cm^−1^). Finally, the average of the triplicate FTIR spectra was obtained. Second derivative and curve fitting of erythrocytes spectra were used to obtain features that occur on spectral peaks and were used to estimate if the features are important in terms of their relationship between control and irradiated groups or in the ability to predict the repair during postirrradiation time.

### Statistical analysis

Data were represented with their mean value ± standard deviation (SD) using ORIGIN 8.0951 software (OriginLab Corporation, Massachusetts, USA). The differences between control and irradiated data were tested using the t-test for independent measurements. P < 0.05 was considered as significant. Hierarchical cluster analysis (HCA) is a simple and rapid procedure that depends on the similarity between two objects [27, 28]. It was used to distinguish between spectra of control and irradiated rats. Using Ward’s algorithm Euclidean distance linkage tree, HCA was used to classify the second derivative of the FTIR spectra into two regions, (3500–2800 cm^−1^) that contains protein, amides A and B, and CH groups of lipid of cell membrane and (1800–1500 cm^−1^) that contains protein, amides I and II. HCA was performed by PAST version 2.17b software [29].

## Results

### FTIR spectral signature

**Fig 1:**
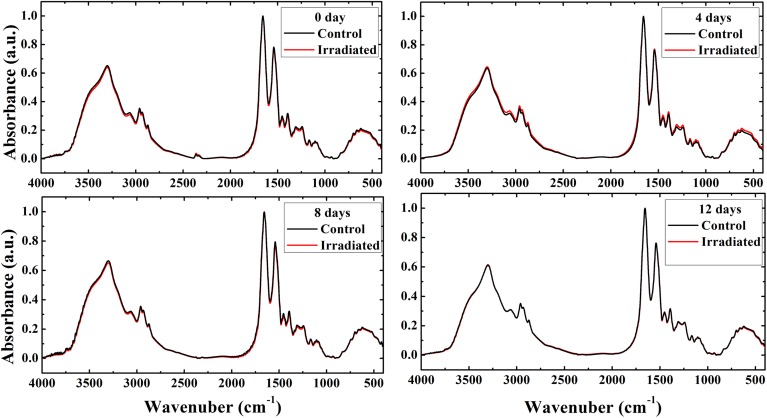
Erythrocytes FTIR spectra. Average of FTIR spectra exhibit rats erythrocytes obtained from the control and irradiated groups at 0, 4, 8, and 12 days postirradiation.

The FTIR spectra of the control and irradiated erythrocytes in the range of 4000–400 cm^−1^ for 0, 8 and 12 days postirradiation groups showed negligible changes and they are nearly superimposed. While the spectrum of the 4 days postirradiation group showed slight changes in the intensity of band and band shift compared to the spectrum of its control (Fig 1).

Table 1 gives the most characteristic bands with their proposed assignment. They were estimated according to the literatures [5, 25, 30–37]. To split the overlaid bands, second derivatives of the FTIR spectra were taken into two distinct regions (3500–3040 cm^−1^) and (3020–2800 cm^−1^).

**Table 1:** Proposed Band assignments of the FTIR spectrum of rat erythrocytes on the 4000–400 cm^−1^ spectral range.

Wave number (cm^−1^)	Band assignment
3294	Amide A: Mainly ν(N-H) of proteins with ν(O-H) of water and protein
3060	Amide B: N-H stretching of proteins
2960	ν_as_(CH_3_): mostly proteins, lipids
2933	ν_as_(CH_2_): mostly lipids, proteins
2871	ν_s_(CH_3_): mostly proteins, lipids
1654	Amide I: ν (C = O), ν (C-N), δ (N-H)
1541	Amide II: δ(N-H) and ν(C-N) of the polypeptide
1452	δ_as_(CH_3_), δ_as_(CH_2_), δ_s_(CH_3_), and δ_s_(CH_2_): Phospholipids, fatty acids, glycerides)
1395	ν_s_ (COO^−^): lipids, proteins (fatty acids and amino acids)
1307	Amide III: band components of proteins
1242	ν_as_(PO_2_^−^): phospholipids
1166	ν_as_(COOC^−^): carbohydrates and proteins
1105–1080	ν_s_(PO_2_^−^): phospholipids
740	ν (N-H) of thymine

Note. ν = bond stretch; s = symmetric vibration; as = asymmetric vibration; δ = bending vibration.

### N-H and OH stretching bands (3500–3040 cm^−1^)

**Fig 2:**
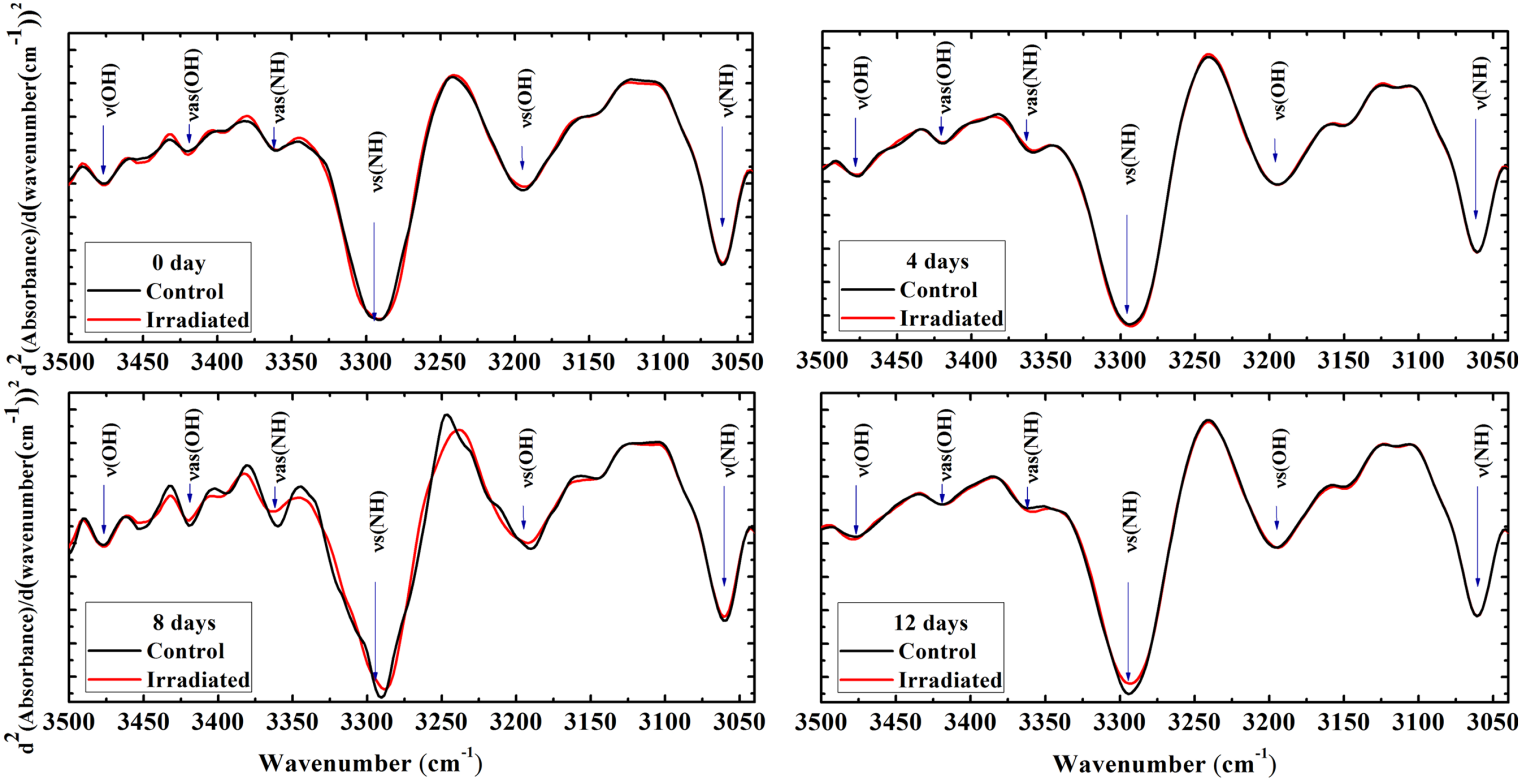
FTIR spectra second derivative of erythrocytes amides A and B. Second derivative of the average of FTIR rat erythrocytes spectra in the range (3500–3040 cm^−1^) obtained from the control and irradiated groups at 0, 4, 8, and 12 days postirradiation. Major bands were ν = (OH), νas(OH), νas(NH), νs(NH), νs(OH), and ν = (NH).

FTIR spectra second derivative in this range revealed six bands, namely ν(NH), ν_s_(OH), ν_s_(NH), ν_as_(NH), ν_as_(OH), and ν(OH) [31, 34] (Fig 2). Table 2 gives the mean values ∓ SD of the integrated area of bands. The data revealed noticeable decreases in the NH and OH stretching band areas in the 0, 8 and 12 irradiated groups' spectra compared to their control groups. In 0 day irradiated group, significant decrease was detected in the amide B area. In addition, significant decreases were recorded in amides A and B of 8 days. In all bands of 4 days groups, marked increases were observed in the irradiated group compared to the control while, significant changes were recorded only in the protein bands (amides A and B) centered at 3294 cm^−1^ and 3061 cm^−1^, respectively.

**Table 2:** Integrated areas and the percent changes of O-H and N-H stretching bands (3500–3040 cm^−1^) of the rat erythrocytes FTIR spectra of control and irradiated groups at 0, 4, 8, and 12 days postirradiation.

**A**						
	**0 Day**			**4 Days**		
**Functional groups**	**Control**	**Irradiated**	**ΔChange** ^**b**^ **(%)**	**Control**	**Irradiated**	**ΔChange** ^**b**^ **(%)**
ν(NH)	4.10 ± 0.38	3.93 ± 0.11^a^	-4.15	4.62 ± 0.06	4.83 ± 0.06^a^	4.55
ν_s_(OH)	4.28 ± 0.45	4.02 ± 0.13	-6.07	5.06 ± 0.06	5.31 ± 0.09	4.94
ν_s_(NH)	8.70 ± 0.62	8.54 ± 0.24	-1.84	9.56 ± 0.11	9.88 ± 0.08^a^	3.35
ν_as_(NH)	1.54 ± 0.16	1.42 ± 0.05	-7.79	1.85 ± 0.01	1.94 ± 0.04	4.86
ν_as_(OH)	2.02 ± 0.21	1.90 ± 0.04	-5.94	2.16 ± 0.04	2.29 ± 0.04	6.02
ν(OH)	3.41 ± 0.30	3.27 ± 0.04	-4.11	3.53 ± 0.04	3.66 ± 0.05	3.68
**B**						
	**8 Days**			**12 Days**		
**Functional groups**	**Control**	**Irradiated**	**ΔChange** ^**b**^ **(%)**	**Control**	**Irradiated**	**ΔChange** ^**b**^ **(%)**
ν(NH)	4.85 ± 0.78	3.91 ± 0.37^a^	-19.38	4.59 ± 0.09	4.49 ± 0.02^a^	-2.18
ν_s_(OH)	5.39 ± 1.05	4.08 ± 0.45^a^	-24.30	5.03 ± 0.12	4.93 ± 0.04	-1.99
ν_s_(NH)	9.79 ± 1.21	8.42 ± 0.63	-13.99	9.55 ± 0.11	9.32 ± 0.04^a^	-2.41
ν_as_(NH)	1.94 ± 0.40	1.51 ± 0.14	-22.16	1.82 ± 0.04	1.80 ± 0.02	-1.10
ν_as_(OH)	2.51 ± 0.43	2.03 ± 0.21	-19.12	2.19 ± 0.07	2.14 ± 0.01	-2.28
ν(OH)	3.97 ± 0.55	3.30 ± 0.27^b^	-16.88	3.58 ± 0.06	3.52 ± 0.02	-1.68

Parameters (mean ± SD)×10^−3^ were obtained by integrating the bands of second derivative of FTIR spectra,

^a^significant change to the control values (p < 0.05),

^b^Δchange (%) are the percent change of irradiated relative to the control.

### CH stretching bands (3020–2800 cm^−1^)

**Fig 3:**
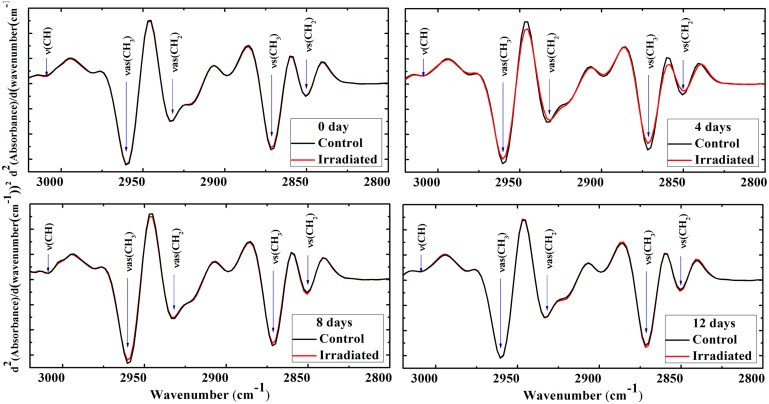
FTIR spectra second derivative of erythrocytes CH stretching bands. Second derivative of the average of FTIR rat erythrocytes spectra in the range 3020–2800 cm^−1^ obtained from the control and irradiated groups at 0, 4, 8, and 12 days postirradiation. Major bands were ν = (CH), νs(CH_3_), νs(CH_2_), νas(CH_3_), and νas(CH_2_).

Fig 3 shows FTIR spectra second derivative in the range 3020–2800 cm^−1^, which is mainly attributed to the olefinic CH stretching, asymmetric, and symmetric stretching of the methyl and methylene groups [11, 30, 33–38]. The spectral behaviors of the bands ν_s_(CH_3_) (2960 cm^−1^), ν_s_(CH_2_) (2933 cm^−1^), ν_as_(CH_3_) (2871 cm^−1^) are very characteristic upon lipid oxidation and can be considered as alternative method for peroxide determinations as the oxidative stress or disordering the hydrocarbon lipid chains. The olefinic band (C = CH) centered at 3010 cm^−1^ is sensitive to the degree of unsaturation fatty acids. Interestingly, this band area increased significantly for only 4 days postirradiated group compared to the its control group (Table 3).

**Table 3:** Integrated areas and the percent changes of C-H stretching bands (3020–2800 cm^−1^) of the rat erythrocytes FTIR spectra of control and irradiated groups at 0, 4, 8, and 12 days postirradiation.

**A**						
	**0 Day**			**4 Days**		
**Functional group**	**Control**	**Irradiated**	**ΔChange** ^**a**^ **(%)**	**Control**	**Irradiated**	**ΔChange** ^**b**^ **(%)**
ν_s_(CH_2_)	08.78 ± 0.07	08.66 ± 0.23	-1.37	07.59 ± 0.36	08.53 ± 0.05^a^	12.38
ν_s_(CH_3_)	15.82 ± 0.18	15.48 ± 0.10^a^	-2.15	14.27 ± 0.10	15.63 ± 0.14^a^	09.53
ν_as_(CH_2_)	21.58 ± 0.19	21.17 ± 0.26	-1.90	19.03 ± 0.41	21.11 ± 0.16^a^	10.93
ν_as_(CH_3_)	19.85 ± 0.17	19.49 ± 0.21	-1.81	17.88 ± 0.27	19.49 ± 0.14^a^	09.00
ν(CH)	08.39 ± 0.05	08.29 ± 0.14	-1.19	07.05 ± 0.18	08.22 ± 0.04^a^	16.60
**B**						
	**8 Days**			**12 Days**		
**Functional group**	**Control**	**Irradiated**	**ΔChange** ^**a**^ **(%)**	**Control**	**Irradiated**	**ΔChange** ^**b**^ **(%)**
ν_s_(CH_2_)	09.23 ± 0.26	08.93 ± 0.13	-3.25	08.15 ± 0.14	08.35 ± 0.08	2.45
ν_s_(CH_3_)	16.22 ± 0.80	15.50 ± 0.14	-4.44	15.26 ± 0.04	15.54 ± 0.07^a^	1.83
ν_as_(CH_2_)	22.29 ± 0.91	21.47 ± 0.24	-3.68	20.52 ± 0.17	20.92 ± 0.04^a^	1.95
ν_as_(CH_3_)	20.43 ± 0.90	19.59 ± 0.21	-4.11	18.88 ± 0.08	19.11 ± 0.03	1.22
ν(CH)	08.87 ± 0.29	08.42 ± 0.08	-5.07	7.88 ± 0.06	07.98 ± 0.03	1.27

Parameters (mean ± SD)×10^−3^ were obtained by integrating the bands of second derivative of FTIR spectra,

^a^significant change to the control values (p < 0.05),

^b^Δchange (%) are the percent change of irradiated relative to the control.

Meanwhile, this olefinic band area showed a slight decrease in its area for 0 and 8 days and a slight increase for 12 days postirradiation without significant changes. On the other hand, a marked and significant increase is noticed in the symmetric and asymmetric stretching of CH_3_ and CH_2_ bands areas for the 4 days irradiated group compared to the control. No changes between irradiated and control groups were noticed in 12 days postirradiation group. For 4 days postirradiated group, the position of the symmetric and the asymmetric stretching CH_2_ absorbance bands shifted toward lower frequencies (Fig 3). The shift in these bands is referred to the changes in membrane fluidity. Frequency becomes lower, as membrane fluidity gets lower [26, 33, 39]. Band shifted significantly towards the lower frequencies for both CH_2_ asymmetric and olefinic CH for 8 days postirradiated group was also detected. However, no band shift was detected in the other tested groups,.

### C = O and C-N stretching and N-H bending (1800–1500 cm^−1^)

The amides I and II bands that centered at 1654 cm^−1^ and 1542 cm^−1^ respectively, are associated with the ν (C = O), ν (C-N), and δ (N-H) modes of protein content in the cells. The wide overlapping of bands in the raw spectrum causes difficulty in band segregation and interpretation of data [40]. Curve fitting methods can characterize all bands of a spectral range that correspond exactly to the molecules present in the sample [31]. This method was applied in the spectra of control and irradiated groups in order to explore the effect of neutron irradiation on the protein secondary structure and the carbonyl formation over the range 1800–1500 cm^−1^. The baseline was in the region: 1800-1480cm^−1^. Relative positions and the number of peaks in the amides I and II bands were determined using second derivative arithmetical function. This region includes the amides II, I, and ester carbonyl over the ranges 1600–1500, 1700–1600, and 1750–1700 cm^−1^, respectively. Figures A and B in S1 File show the best curve fitting for the amides I, II, and ester carbonyl bands contour of all the tested groups. Amide I bands are used for quantitative secondary determinations, whereas amide II bands are rarely to use. Table 4 summarizes the positions, fractional percentage areas, and band assignment of the amide I components for all the tested groups. The bands over the ranges [1614–1619 cm^−1^], [1624–1640 cm^−1^], [1642–1646 cm^−1^], [1652–1659 cm^−1^], and [1660–1674 cm^−1^] are attributed to the β-turn, β-structure, random coil, α-helix structure, and the β-turn, respectively. While the bands over the range [1676–1686 cm^−1^] and [1693–1698 cm^−1^] are attributed to the parallel and anti-parallel β-strand, respectively [31, 32, 35, 40–44]. It is obvious that all protein sub-band areas significantly increased or decreased in irradiated group compared to the control on the 4 days postirradiation (Table 4).

**Table 4:** Percentage areas of protein secondary structures and their band assignments of rat erythrocytes FTIR spectra of the control and irradiated groups at 0, 4, 8, and 12 days postirradiation.

**A**					
		**0 Day**		**4 Days**	
**Wavenumber (cm** ^**−1**^ **)**	**Band Assignment**	**Control**	**Irradiated**	**Control**	**Irradiated**
1614–1619	β-turn	07.58 ± 0.89	07.64 ± 0.98	06.68 ± 0.45	02.83 ± 0.33^a^
1624–1640	β-Structure	10.76 ± 0.75	10.40 ± 1.09	11.41 ± 1.75	18.30 ± 3.35^a^
1642–1646	random coil	15.24 ± 0.87	13.60 ± 0.65^a^	14.40 ± 2.05	17.63 ± 1.09^a^
1652–1659	α-helix	16.96 ± 1.51	14.68 ± 0.98	16.96 ± 1.95	25.48 ± 2.13^a^
1660–1674	β-turn	21.95 ± 3.72	29.74 ± 3.79^a^	17.58 ± 4.04	16.20 ± 1.19
1676–1686	Parallel β-strand	15.96 ± 1.88	13.79 ± 3.62	17.25 ± 1.11	10.83 ± 1.98^a^
1693–1698	Anti-parallel β-strand	11.54 ± 1.36	10.15 ± 1.23	16.28 ± 3.24	08.73 ± 3.39^a^
**B**					
		**8 Days**		**12 Days**	
**Wavenumber (cm** ^**−1**^ **)**	**Band Assignment**	**Control**	**Irradiated**	**Control**	**Irradiated**
1614–1619	β-turn	05.99 ± 0.79	06.32 ± 1.37^a^	02.78 ± 3.08	03.02 ± 1.09^a^
1624–1640	β-Structure	16.02 ± 3.27	15.16 ± 2.75	17.54 ± 2.26	17.20 ± 1.06
1642–1646	random coil	11.23 ± 2.65	11.37 ± 1.09	12.92 ± 2.17	12.45 ± 1.15
1652–1659	α-helix	13.49 ± 1.91	13.50 ± 1.89	15.55 ± 1.92	16.46 ± 3.07
1660–1674	β-turn	29.04 ± 5.03	23.96 ± 3.39	19.23 ± 2.03	22.87 ± 2.63^a^
1676–1686	Parallel β-strand	13.78 ± 1.09	20.54 ± 4.09^a^	22.49 ± 2.27	15.67 ± 4.61
1693–1698	Anti-parallel β-strand	10.46 ± 0.78	09.15 ± 0.67	09.50 ± 0.59	12.34 ± 3.13

Parameters (mean ± SD) were obtained by curve fitting of FTIR spectra,

^a^ significant change to the control values (p < 0.05).

**Fig 4:**
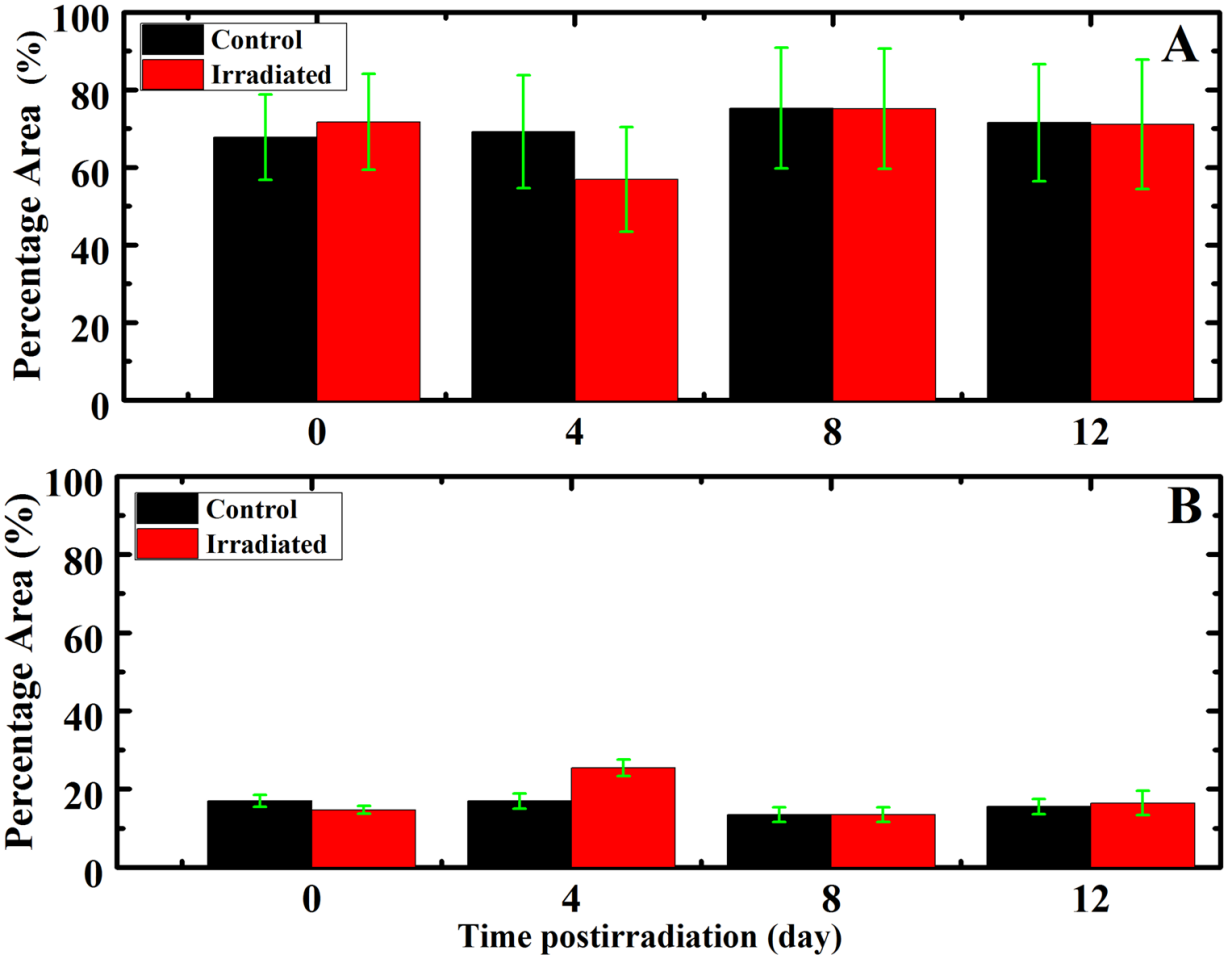
Percentage areas of protein secondary structure as a function of the postirradiation time. (A) Percentage areas of β-structure (B) Percentage areas of α -helix.

The most important differences between control and irradiated groups at 4 days postirradiation were in the dramatic increase in the α-helix protein structure opposing the severe decrease in the β structure (Fig 4). Moreover, there is a significant decrease in the anti-parallel β-structure in the irradiated group compared to the control group. It should be mentioned that the total area of amide II bands of control and all irradiated groups remain unchanged. While amide I bands total areas increase only in the 0 day irradiated group. On the other hand, this band area is drastically decreased for the 4 days irradiated groups compared to the control. Thus, the changes in the protein content of erythrocytes refer only to the changes in the amide I rather than to the amide II.

**Fig 5:**
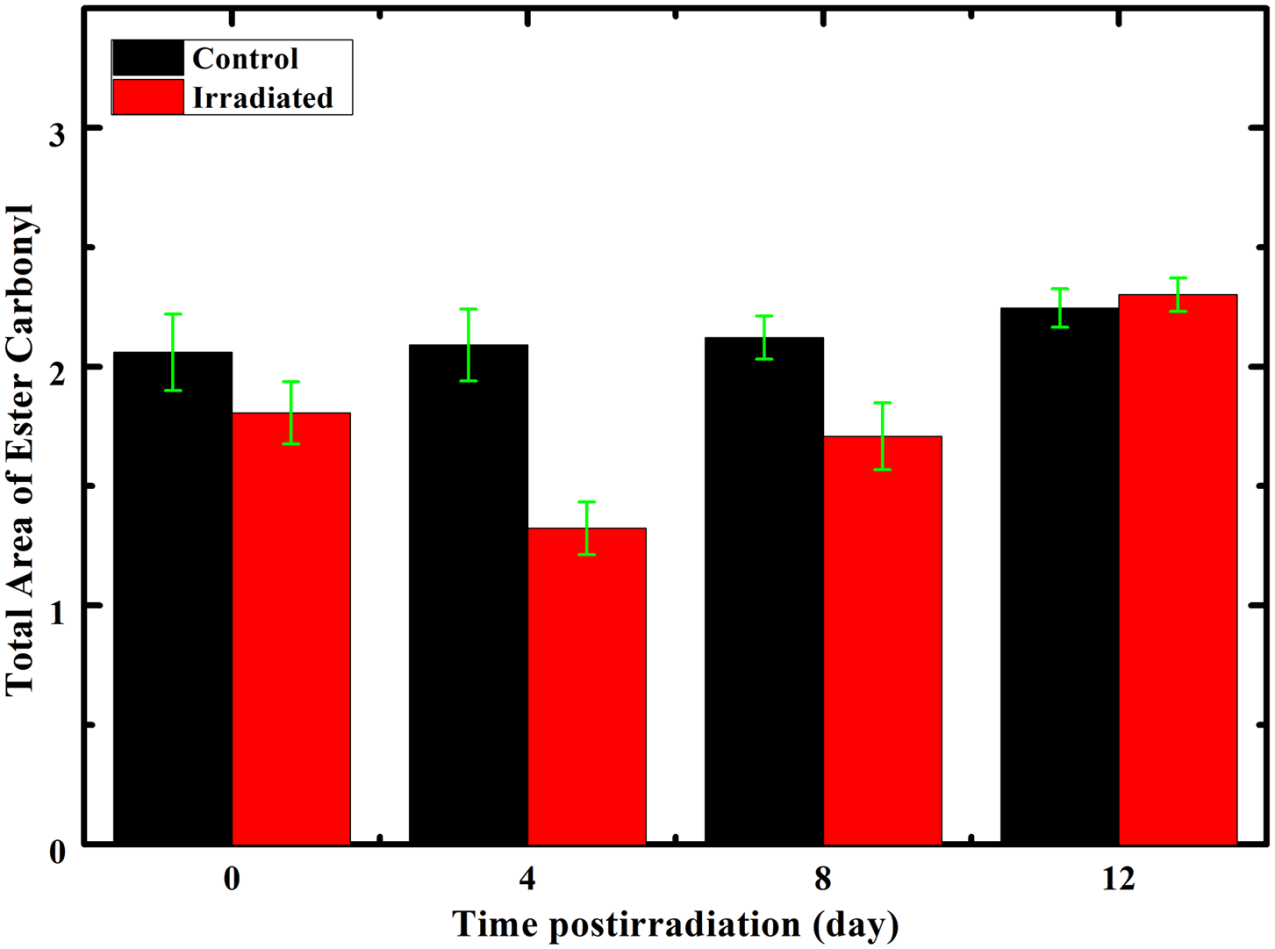
Area of ester sub-bands C = O associated with phospholipids. The total area of ester sub-bands C = O associated with phospholipids in FTIR rat erythrocytes spectra of the control and irradiated groups as a function of the postirradiation time.

The total areas of the ester carbonyl sub-bands from both control and irradiated groups are illustrated in Fig 5. It is obvious that the total area of these bands decreased in all irradiated groups at 0, 4, and 8 days postirradiation compared to the control groups. The minimum point was detected in the 4 days irradiated group. While, there was no change between the control and the irradiated groups at 12 days postirradiation.

### Clustering analysis

**Fig 6:**
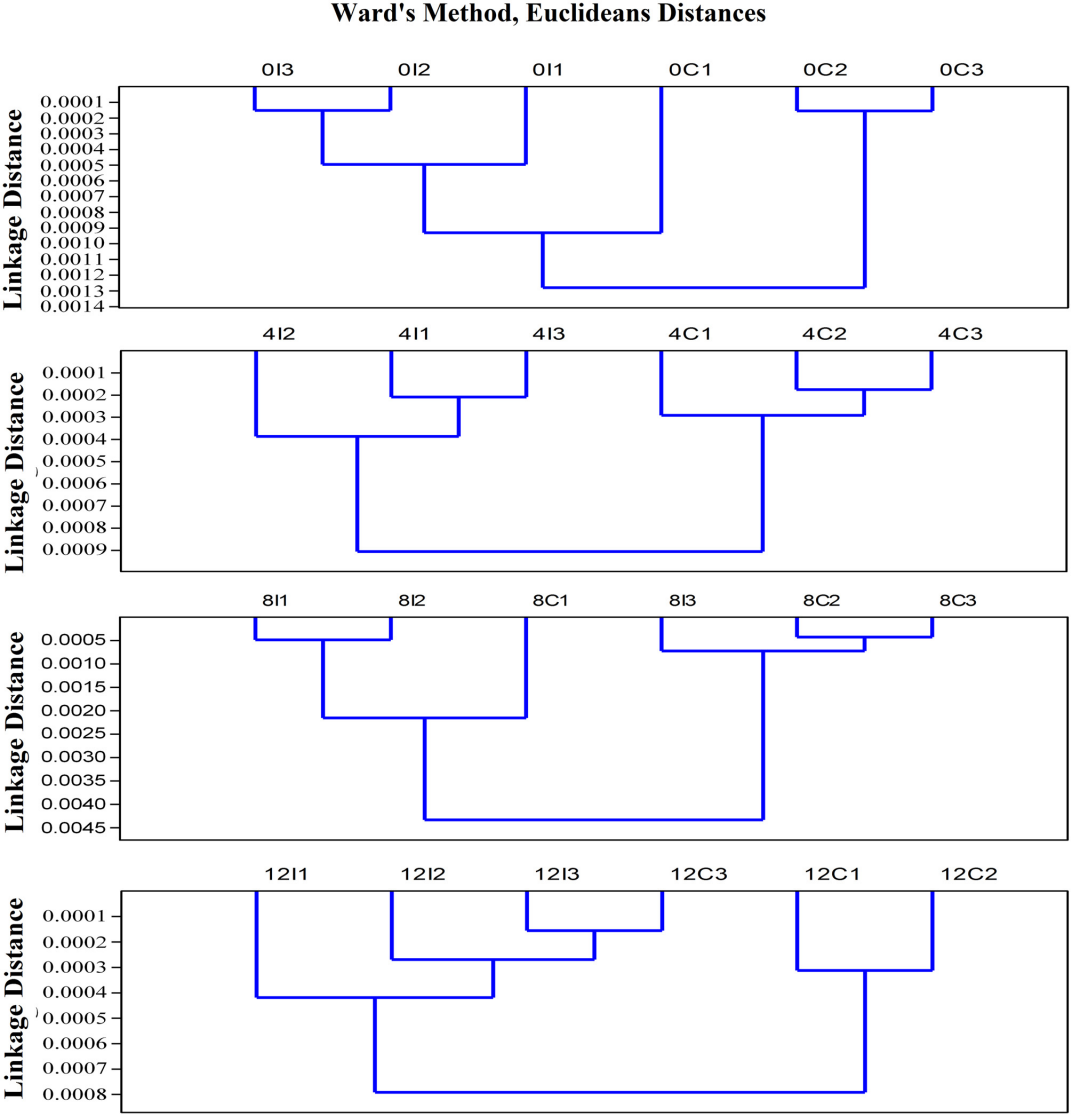
HCA of rats' erythrocytes Protein and cell membrane FTIR spectra. HCA spectral analysis of protein and carbonyl (C = O) (1800–1500 cm^−1^); CH_3_, CH_2_ asymmetric and symmetric stretching, amid A, and amid B (3500–2800 cm^−1^) of rat erythrocytes obtained from the control and irradiated groups at 0, 4, 8, and 12 days postirradiation.

The dendrograms of HCA created two initial clusters represented by two branches, which were further subdivided into smaller clusters (Fig 6). Wards method Euclidean distances dendrogram, linkage tree, succeeded to distinguish between irradiated and control rats at 4 days postirradiation. However, it failed to distinguish between irradiated and control rats in other groups.

### Curve fitting of 4 days (3500–2800 cm^−1^)

According to the previous results, the statistical significance methods and the HCA that showed the most significances and changes were detected at 4 days postirradiation. Hence, the spectra of 4 days groups were reanalyzed using curve fitting in the region of 3500–2800 cm^−1^. This region includes CH_3_, CH_2_ asymmetric and symmetric stretching, and amides A and B. Thus, the reanalyzing was achieved to discover the membrane perturbation and protein denaturation due to VLDFN in erythrocytes at 4 days postirradiation. Then, the ratio measurements were calculated.

Table 5 represents the peak position, height, full width at the half-height (FWHH), and area of CH_3_, CH_2_ asymmetric and symmetric stretching, and amides A and B of erythrocytes obtained from control and irradiated groups on 4 days postirradiation. It is apparent from the table that the CH_2_ symmetric and asymmetric stretching bands showed marked bands broadening with increasing bands area for the irradiated group compared to the control group. By contrast, there are noticeable decreases in these band areas in addition to FWHH of CH_3_ symmetric and asymmetric stretching bands. It can be seen that there was shift in the amide A band to lower frequency (Table 5).

**Table 5:** CH_3_, CH_2_ asymmetric and symmetric stretching, amides A and B (3500–2800 cm^−1^) of rats' erythrocytes sub-bands of control and irradiated groups at 4 days postirradiation. Wavenumbers, height of peaks, FWHHs, and areas corresponding to the resultant curve fitted Gaussian peaks.

Group	Assignments	Band center	Height	FWHH	Area
Control	ν_s_(CH_2_)	2850.71 ± 0.11	0.021 ± 0.005	87.26 ± 5.07	2.21 ± 0.59
Control	ν_s_(CH_3_)	2871.41 ± 0.01	0.052 ± 0.003	44.85 ± 1.88	2.21 ± 0.22
Control	ν_as_(CH_2_)	2932.63 ± 0.04	0.103 ± 0.001	67.74 ± 0.60	7.53 ± 0.08
Control	ν_as_(CH_3_)	2960.24 ± 0.04	0.091 ± 0.009	54.55 ± 1.30	5.26 ± 0.20
Control	ν(CH)	3009.72 ± 1.04	0.012 ± 0.009	76.25 ± 3.95	0.32 ± 0.09
Control	Amide B	3061.88 ± 0.07	0.080 ± 0.003	96.42 ± 8.30	8.56 ± 0.79
Control	Amide A	3298.33 ± 7.07	0.251 ± 0.030	103.22 ± 9.11	28.20 ± 0.22
Irradiated	ν_s_(CH_2_)	2849.38 ± 0.00	0.028 ± 0.007	81.19 ± 14.84	2.49 ± 0.90
Irradiated	ν_s_(CH_3_)	2871.21 ± 0.01	0.043 ± 0.001	44.64 ± 15.79	2.07 ± 0.76
Irradiated	ν_as_(CH_2_)	2931.67 ± 0.01	0.093 ± 0.023	69.29 ± 8.68	7.08 ± 0.56
Irradiated	ν_as_(CH_3_)	2959.93 ± 0.03	0.099 ± 0.021	59.04 ± 7.06	6.38 ± 0.95
Irradiated	ν(CH)	3008.72 ± 0.29	0.004 ± 0.001	72.58 ± 10.64	0.64 ± 0.11
Irradiated	Amide B	3061.00 ± 0.15	0.094 ± 0.013	87.97 ± 6.45	8.74 ± 0.72
Irradiated	Amide A	3291.67 ± 5.04	0.229 ± 0.065	109.3 ± 18.17	27.81 ± 4.73

Parameters are expressed as the mean ± SD.

### Ratios measurement

Area ratios of some specific infrared bands have been evaluated according to the reports in the literatures [5, 32, 38, 45–52], which were used for quantitative comparison between the control and irradiated groups at 4 days postirradiation (Table 6). The ratios were calculated using the curve fitted FTIR data. The ratios ν_as_(CH_2_)/ν_s_(CH_3_), ν_s_(CH_2_)/ν_s_(CH_3_), ν_as_(CH_2_)/ν_as_(CH_3_), and ν_as_(CH_2_)/total lipids were calculated in order to investigate the membrane polarity, lipid chain packing, the degree of saturation, and phospholipids chain length, respectively. The data revealed a noticeable increase in the erythrocytes membrane polarity and packing on 4 days postirradiated group compared to the control group.

**Table 6:** Ratios characteristics of the biomarkers derived from rat erythrocytes FTIR spectra of control and irradiated group at 4 days postirradiation.

Ratio	Indicator	Control	Irradiated
ν(CH) /ν_as_(CH3)	Unsaturated phospholipids	0.06 ± 0.017	0.10 ± 0.023
ν_as_(CH_2_)/ν_as_(CH_3_)	Saturation level or fatty acid chain length	1.43 ± 0.057	1.11 ± 0.187
ν_s_(CH_2_)/ν_s_(CH_3_)	Lipid chains packing	1.00 ± 0.284	1.20 ± 0.620
ν_as_(CH_2_) /ν_s_(CH_3_)	Bilayer order or disorder lipid	3.40 ± 0.346	3.42 ± 1.283
ν_as_(CH_2_)/total lipids	Hydrocarbon chain length	0.77 ± 0.120	0.74 ± 0.275
C = O/Amide II	Carbonyl formation against lipase action	0.36 ± 0.065	0.21 ± 0.037
Amide II/amide I	Change composition of the protein pattern	0.80 ± 0.164	0.58 ± 0.107
Amide A/amide B	Protein pattern	3.29 ± 0.612	3.18 ± 0.543
C = O /total lipids	Carbonyl status of the system	0.21 ± 0.052	0.14 ± 0.051
Total lipids/total proteins	Lipids to proteins	0.74 ± 0.119	0.56 ± 0.112

Parameters are expressed as the mean ± SD.

Neutron irradiation caused also increasing in the hydrocarbon chain length indicated by the low value of the νas(CH_2_)/total lipids ratio in the irradiated group (0.74) compared to the control group (0.77). Meanwhile, dramatic increase in the degree of chain lipid unsaturated from 0.06 in the control to 0.1 in the irradiated group was observed. C = O/amide II and C = O/total lipids ratios determine the degree of the carbonyl formation against lipase action and show the carbonyl status of the system, respectively. These ratios showed a marked decrease in the irradiated sample compared to the control. The changes in the protein secondary structure folding and unfolding was evident by the significant decrease in the amide II/amide I ratio.

## Discussion

RBE of incident neutrons on the tissue is strongly dependent on neutron energy (neutron velocity). Nearly when the energy of neutron beam ranges between 1 eV and 1 MeV, the density of ionization in the tissues increases as the velocity of the neutrons increases. However, the density of ionization in the tissues decreases as the velocity of the neutron increases when the energy of neutron beam is greater than 1 MeV [53]. FTIR spectroscopy has been widely used to study the conformational order of phospholipids in the erythrocytes cell membrane [54] and to investigate the secondary structure of hemoglobin [42] by the influence of certain diseases. For this purpose, the area of the FTIR spectra second derivative and the peak resolved sub-bands of the absorbance's belonging to the main groups of phospholipids CH_3_, CH_2_ symmetric and asymmetric, ester carbonyl C = O, and the protein bands were recorded. The data of second derivative of FTIR spectra were separated into control and postirradiated classes using HCA. It used second derivative FTIR spectral data of the regions 3500–2800 cm^−1^ and 1800–1500 cm^−1^ for classification primarily reflecting differences in phospholipids and proteins. This approach succeeded to separate only the spectra of control rats from that of irradiated rats at 4 days postirradiation, which differ in phospholipids, proteins content, and structure due to neutron irradiation. Consequently, we will focus in this discussion mainly on those groups have been tested at 4 days postirradiation. Selim *et al*. [11] found that gamma radiation causes an increase in the free radicals density in the irradiated erythrocytes and it was a dose-dependent when studied by electron paramagnetic resonance (EPR). Normally, erythrocytes are equipped with a set of antioxidant enzymes that fight against oxidative stress and the membrane lipids compose approximately 40% of the erythrocytes membrane mass [11]. The first detected decrease in the second derivative olefinic band(C = CH) area centered at 3010 cm^−1^ for the 0 days postirradiation group may be attributed to an initial oxidative damage of the erythrocyte membrane lipids [34]. On the other hand, the detected increase in the olefinic band (C = CH) area centered at 3010 cm^−1^ which is sensitive to the degree of unsaturation fatty acids only for 4 days postirradiated group may indicate that fatty acids in the hydrocarbon chain lipid are highly unsaturated. The reason for the increase in this band may be due to lipid peroxidation [55]. These products, above a threshold concentration, are released into the extra or intracellular site of the cell, causing apoptosis and a decrease in the olefinic band intensity of erythrocyte ghosts upon lipid peroxidation [56, 57]. The observed shift in the ν_s_(CH_2_) and ν_as_(CH_2_) bands towards the lower frequencies also suggest the decrease in the erythrocytes membrane fluidity and hence an increase in the membrane conformational order [25, 32, 40]. This is in agreement with the findings of Moore *et al*. [58]. Selim *et al*. [11] reported that erythrocytes methylene stretching modes in the second derivative spectra around 2850, 2920 cm^−1^ could be used as qualitative indicator of acyl chain order in lipid and membrane bilayers. These bands showed a shift towards the lower frequencies in both second derivative and peak resolved spectra for irradiated group at 4 days postirradiation only. Although, the results of Selim *et al*. [11] showed a decrease in the membrane order indicated by the CH_2_ band shift. By contrast, they recorded an increase in the conformational disorder at high doses. Change in membrane permeability and slower rupture rate in erythrocyte membrane when γ- irradiated in low doses (0.01–0.3Gy) was suggested by [35]. The detected increase in the FWHH of the ν_as_(CH) for irradiated group at 4 days postirradiation indicates an increase in the vibrational motion. By contrast, the decrease in the ν_s_(CH) vibrational frequency indicates an environmental changes [11]. The increase of Van der Waals forces that was indicated by the increase in ν_as_(CH_2_)/ν_s_(CH_3_) ratio. The recorded νs(CH_2_)/νs(CH_3_) and νas(CH_2_)/total lipid area ratios revealed increase in the membrane packing and decrease in the length of the membrane hydrocarbon tail of the fatty acids, respectively. These results are in consistent with those reported by Inouye *et al*. [59]. They strongly suggested that the membrane phospholipids of erythrocytes underwent reactive oxygen species (ROS) attacks during oxidative stress, which primarily affected saturation level of fatty acyl chains and phospholipids structure. Such alteration in conformational orders and membrane phospholipid domains may be primarily due to the peroxidative damage [17]. In the present work, the observed peroxidative damage is a result of the neutron irradiation. The mentioned decrease in the ester carbonyl sub-bands total area in the all irradiated groups together with the calculated decrease in both C = O/amide II and C = O/total lipid ratios may be attributed to the addition of carbonyl groups to amino acid residues [48, 58]. Consequently, this may lead to oxidative protein damage since proteins are most susceptible to ROS attacks during oxidative stress [4, 60]. Rodríguez-Casado *et al*. [48] detected a decrease in the C = O/amide II ratio, which is indicative of the weight of formation of carbonyl compounds against lipase action or lipid degradation during lipid oxidation on brain tissue stressed with amphetamine [48]. Therefore, the decrease in this area ratio upon neutron irradiation may suggest that lipid degradation or lipase action predominates over carbonyl formation in erythrocyte cell membrane in addition to the hemoglobin denaturation. The marked decrease in the total lipid to total protein ratio may also support the above-mentioned suggestion. Erythrocyte proteins are mainly hemoglobin for about 90% and spectrin for about 10% [5]. The observed dramatic increase in the α-helix protein structure opposing the severe decrease in the β-structure, marked decrease in the amide II/amide I, and significant decrease in the random coil and parallel and anti-parallel β-pleated sheets structure may be due to the denaturation of proteins in response to the oxidative stress by neutron irradiation.

## Conclusion

The changes in the cell membrane: decrease in the degree of hydrocarbon chain unsaturation, increase in the membrane fluidity and disordering, decreasing in the Van der Waal forces due to the decrease in the membrane polarity, and packing and hydrocarbon chain length may be attributed to ROS attacks during oxidative stress. From these FTIR spectral markers, the present study concluded that VLDFN (0.009 Gy) could induce oxidative stress and damage to the erythrocytes cell membrane. In addition, most of neutron effect on cell membrane appeared in the methyl and methylene groups, which are made up of carbon and hydrogen elements. Hydrogen has small mass and large cross section for fast neutron reaction. Thus, the parts of cells that are composed from light elements would have greater damage during irradiation by fast neutron. From the significant changes in the percentage of α-helix and β-structure in protein, which were detected at 4 days postirradiation, it could be concluded that VLDFN may cause protein denaturation. Moreover, since most significant alterations in the erythrocytes cell membrane and protein secondary structure between control and irradiated groups appeared at 4 days postirradiation. Thus, this study deduced that the alterations in erythrocytes might be due to the indirect effect of the neutron irradiation. Significance changes between the irradiated and control groups at 8 and 12 days postirradiation were fewer than those at 4 days postirradiation were. This indicates that the repair could happen after four days neutron postirradiation.

## Supporting Information

S1 File Curve fitting of average of FTIR rat erythrocytes spectra. Curve fitting of average of FTIR rat erythrocytes spectra in the range 1800–1480 cm^−1^ obtained from the control and irradiated groups at 0 and 4 days postirradiation **(Figure A)**. Curve fitting of average of FTIR rat erythrocytes spectra in the range 1800–1480 cm^−1^ obtained from the control and irradiated groups at 8 and 12 days postirradiation **(Figure B)**.
(DOCX)
