# Supplementary material for: Effects of Very Low Dose Fast Neutrons on Cell Membrane And Secondary Protein Structure in Rat Erythrocytes
Source: PLoS One. 2015 Oct 5;10(10):e0139854. doi: 10.1371/journal.pone.0139854 (PMC4593584; doi:10.1371/journal.pone.0139854)
Supplement: S1 File — Curve fitting of average of FTIR rat erythrocytes spectra in the range 1800–1480 cm−1 obtained from the control and irradiated groups at 0 and 4 days postirradiation (Figure A). Curve fitting of average of FTIR rat erythrocytes spectra in the range 1800–1480 cm−1 obtained from the control and irradiated groups at 8 and 12 days postirradiation (Figure B). (DOCX) [file pone.0139854.s001.docx]

**SUPPORTING INFORMATION**


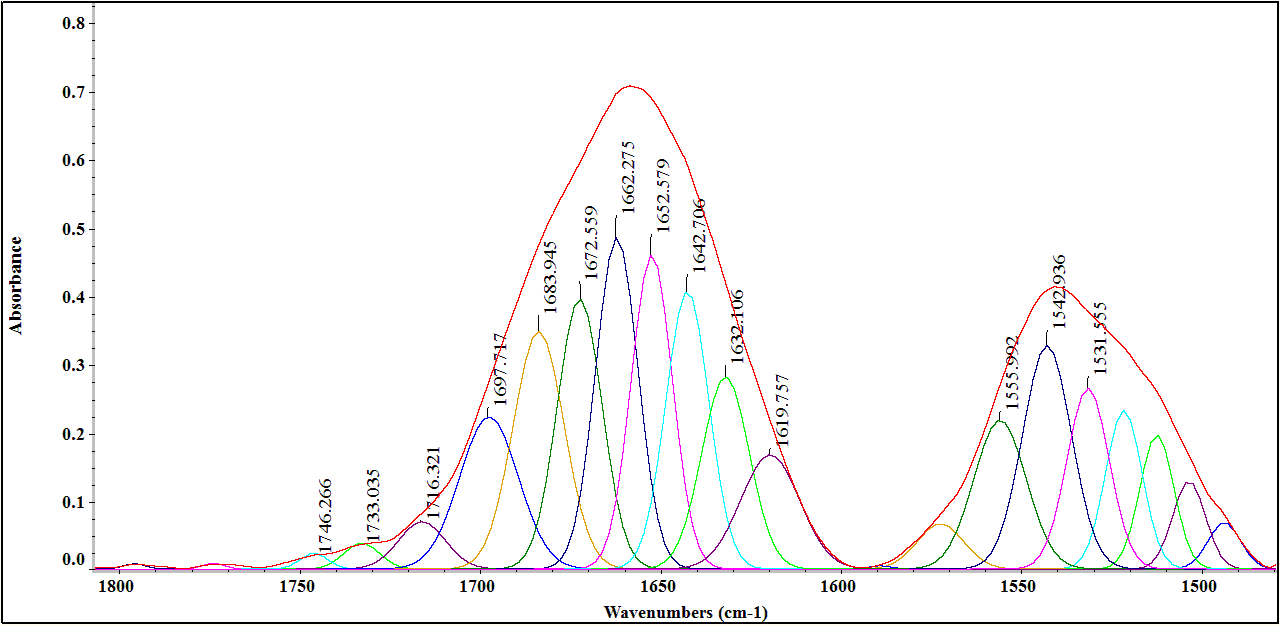

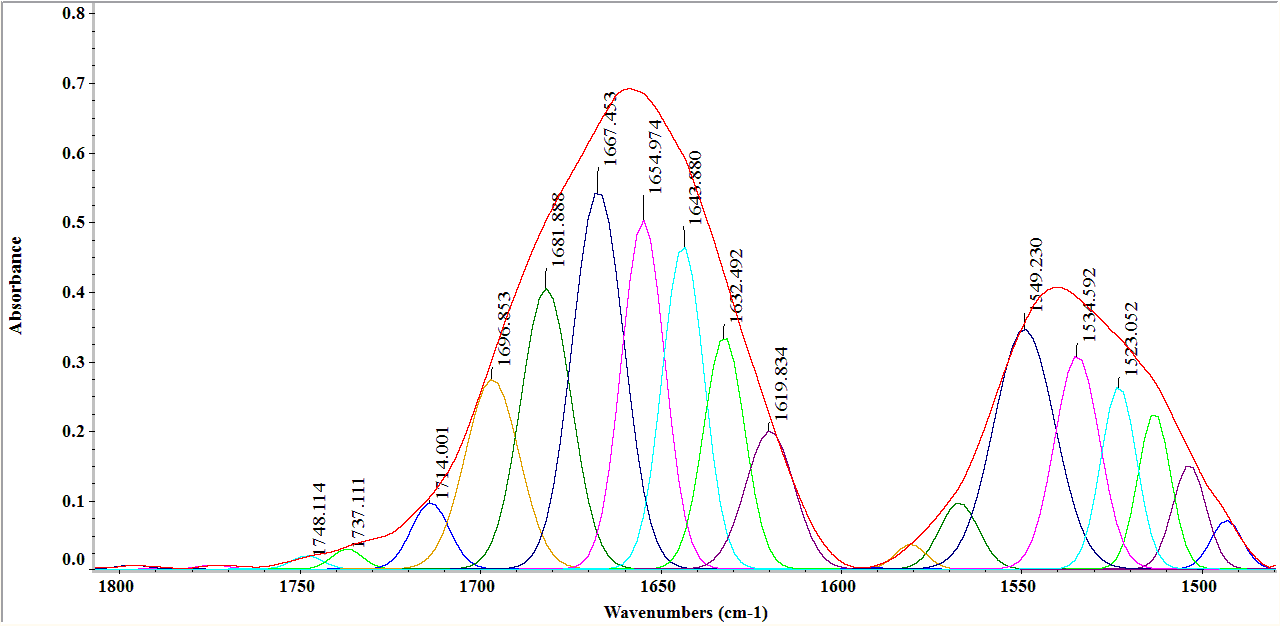

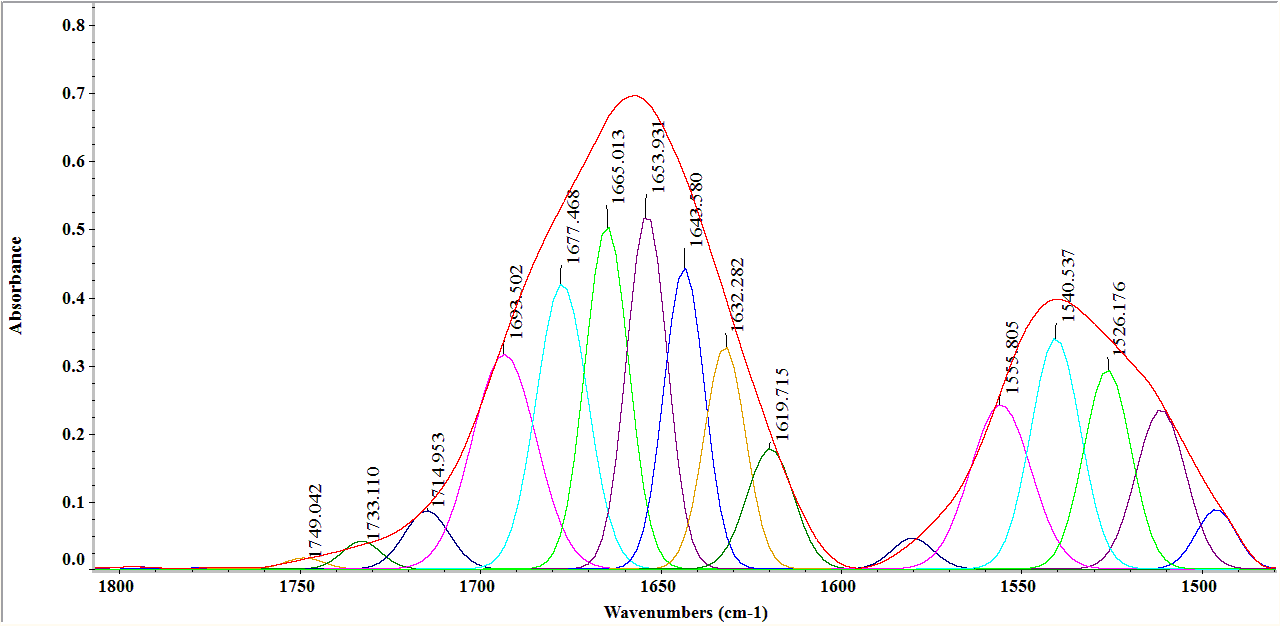

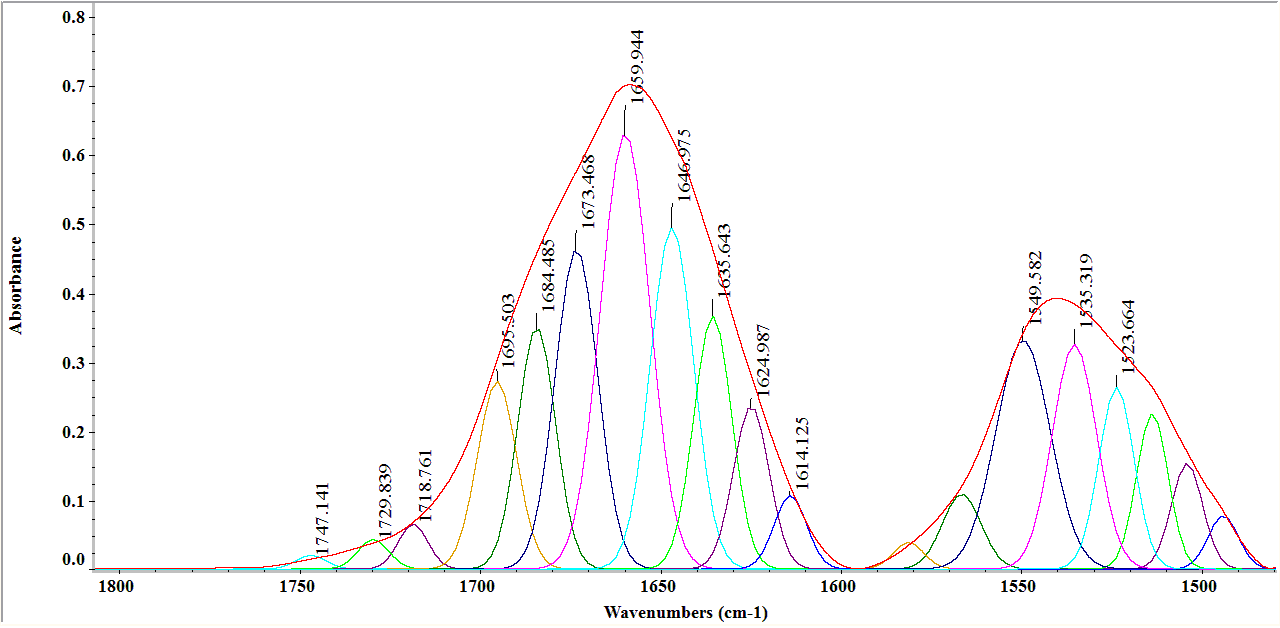


4 days

Irradiated

4 days

Control

0 day

Irradiated

0 day

Control

**Figure A.** Curve fitting of average of FTIR rat erythrocytes spectra in the range 1800-1480 cm^-1^ obtained from the control and irradiated groups at 0 and 4 days postirradiation.


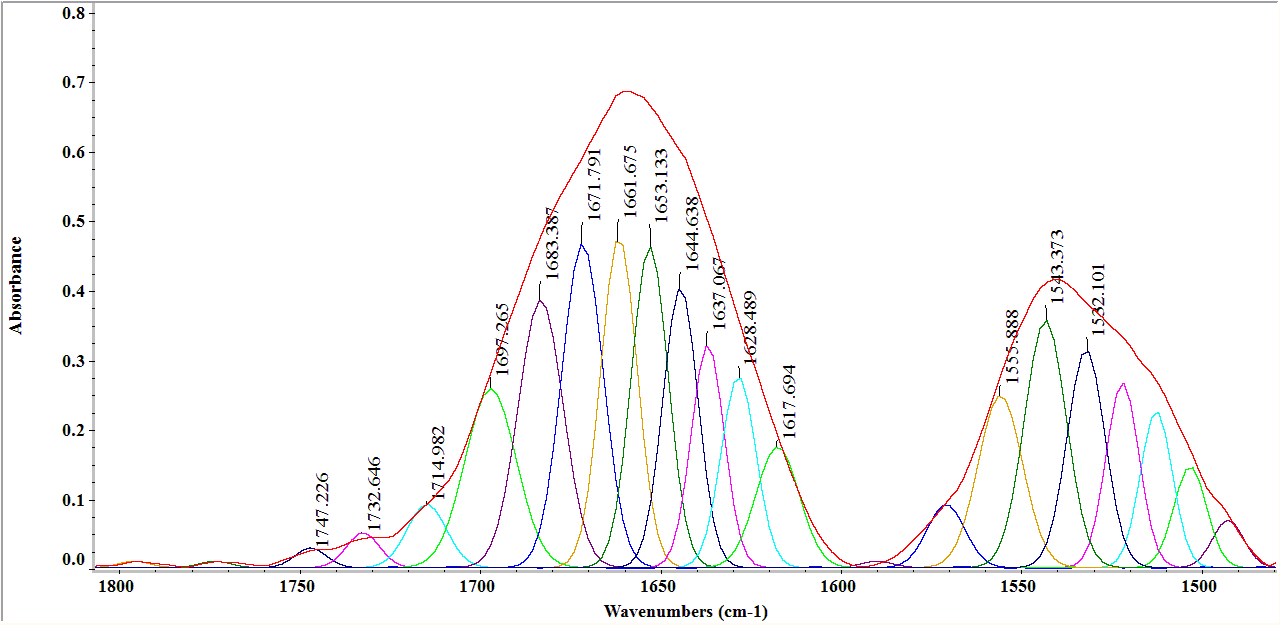

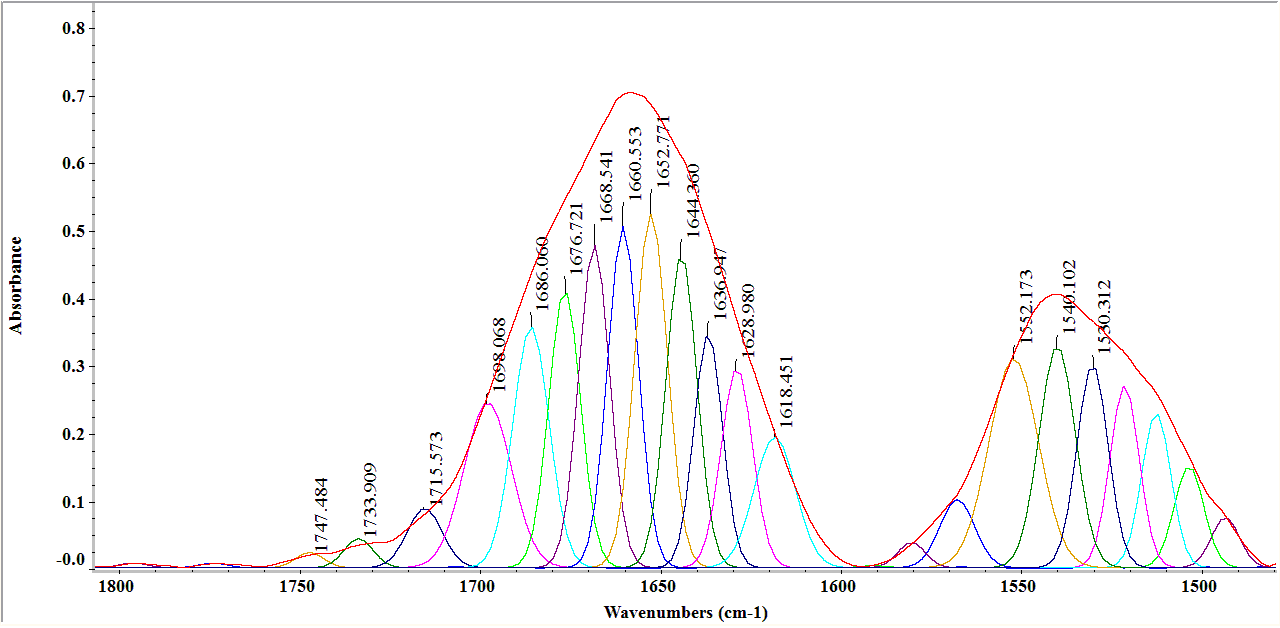

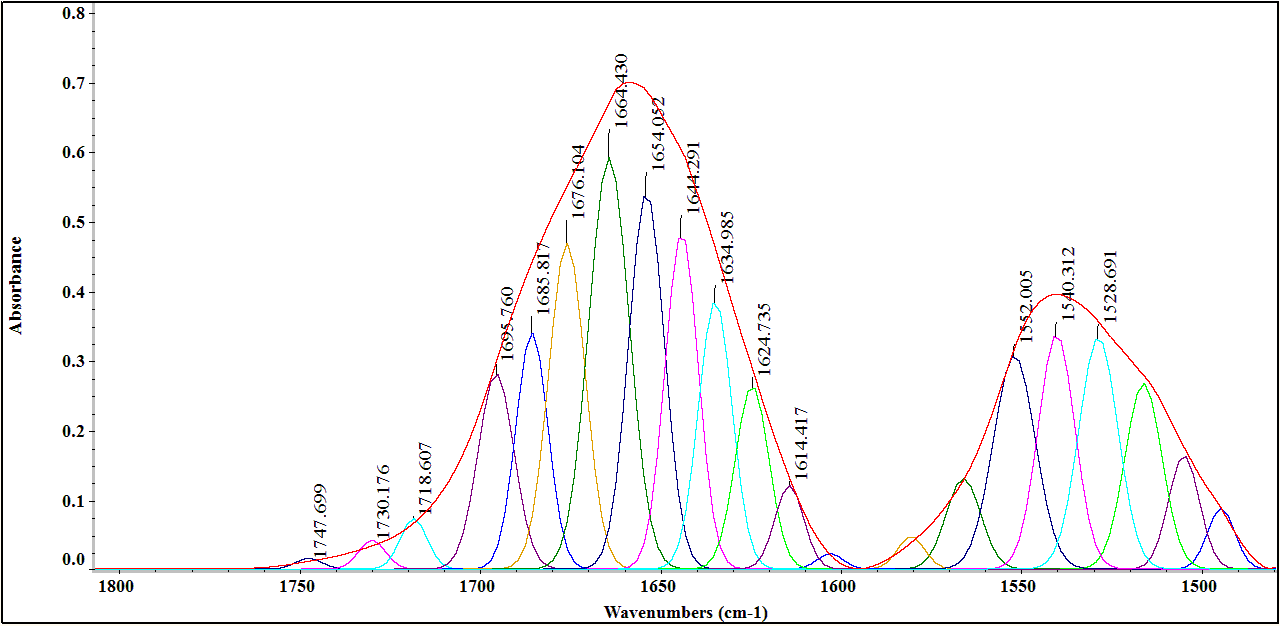

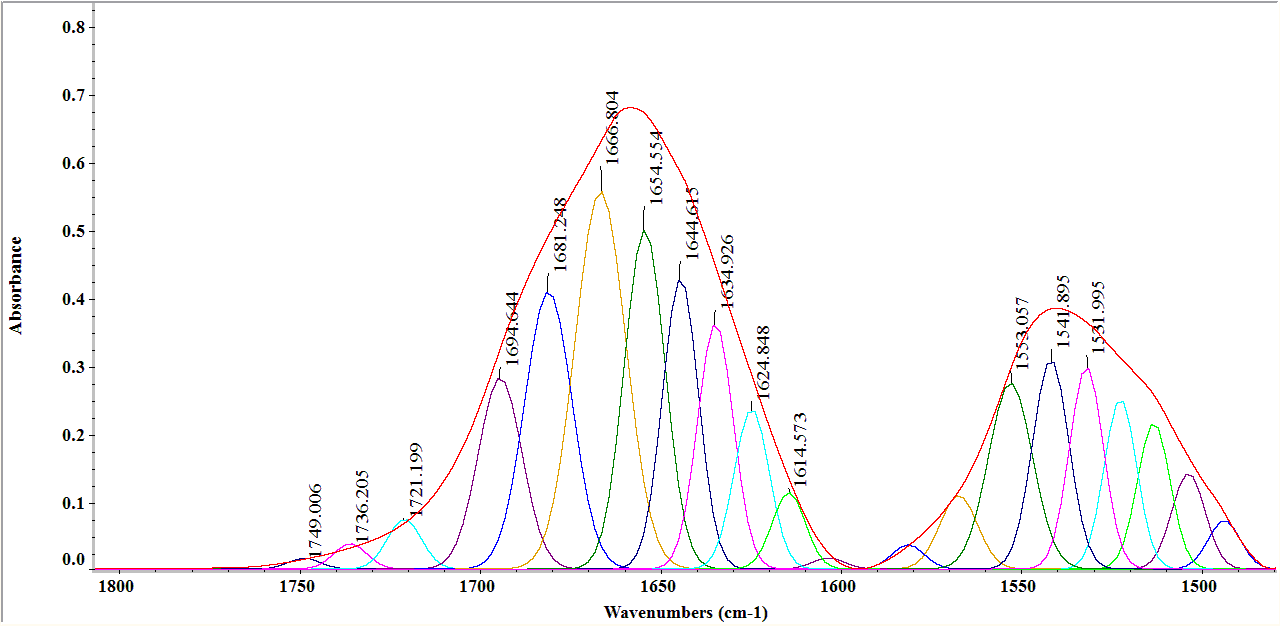


12 days

Irradiated

12 days

Control

8 days

Irradiated

8 days

Control

**Figure B.** Curve fitting of average of FTIR rat erythrocytes spectra in the range 1800-1480 cm^-1^ obtained from the control and irradiated groups at 8 and 12 days postirradiation.
